# Surface Kinetic Mechanisms of Epitaxial Chemical Vapour Deposition of 4H Silicon Carbide Growth by Methyltrichlorosilane-H_2_ Gaseous System

**DOI:** 10.3390/ma15113768

**Published:** 2022-05-25

**Authors:** Botao Song, Bing Gao, Pengfei Han, Yue Yu

**Affiliations:** The Institute of Technological Sciences, Wuhan University, Wuhan 430072, China; 2020106520027@whu.edu.cn (B.S.); 2020106520028@whu.edu.cn (P.H.); 2019106520025@whu.edu.cn (Y.Y.)

**Keywords:** silicon carbide, kinetic mechanism, chemical vapour deposition, numerical model, MTS-H_2_

## Abstract

The chemical vapour deposition (CVD) technique could be used to fabricate a silicon carbide (SiC) epitaxial layer. Methyltrichlorosilane (CH_3_SiCl_3_, MTS) is widely used as a precursor for CVD of SiC with a wide range of allowable deposition temperatures. Typically, an appropriate model for the CVD process involves kinetic mechanisms of both gas-phase reactions and surface reactions. Here, we proposed the surface kinetic mechanisms of epitaxial SiC growth for MTS-H_2_ gaseous system where the MTS employed as the single precursor diluted in H_2_. The deposition face is assumed to be the Si face with a surface site terminated by an open site or H atom. The kinetic mechanisms for surface reactions proposed in this work for MTS-H_2_ gaseous system of epitaxial growth of SiC by CVD technique from mechanisms proposed for H-Si-C-Cl system are discussed in detail. Predicted components of surface species and growth rates at different mechanisms are discussed in detail.

## 1. Introduction

Silicon carbide (SiC) is a very promising material for its resistance to high temperatures and corrosive chemical atmospheres due to its large bandgap, high thermal conductivity, and other unique physical and chemical characteristics [1,2,3,4,5,6]; it can be employed in a variety of applications, such as semiconductor devices, ceramic matrix composites, and aerospace industry [7,8,9,10].

The chemical vapour deposition (CVD) technique shows its unique advantages in epitaxial growth process of SiC [11] since the CVD method is a powerful manufacturing technique for the fabrication of various thin films [12]. The selection of precursors is very critical to the CVD process. SiH_4_-C_3_H_8_-H_2_ gaseous system was widely used in CVD process of SiC [13,14,15], in which silane (SiH_4_) and propane (C_3_H_8_) as precursors, while hydrogen (H_2_) as carrier gas. Halide contained precursors have become preferred because the addition of halogen can modify the CVD process for growing SiC epitaxial layer at relatively high deposition rates [16,17,18,19,20,21]. The chlorinated compounds, available in high purity at low cost [22], are appropriate for SiC epitaxial process. With the addition of HCl as a precursor is a viable approach [22,23,24]. Chlorinated Si-containing compounds such as SiHCl_3_ and SiCl_4_ [19,25] also can be used as precursors.

Besides, an alternative chlorinated compound, methyltrichlorosilane (CH_3_SiCl_3_, MTS), which contains not only Si and C but also Cl, is commercially used as a precursor for silicon carbide with a wide range of allowable deposition temperatures [26,27,28,29,30], and its decomposition is catalyzed by hydrogen, the carrier gas [31]. MTS will decompose in the CVD reactor to form intermediate species containing silicon, carbon and chlorine, and some of these intermediate species contribute greatly to form the SiC film by participating in surface reactions on the substrate.

Extensive experimental investigations performed on SiC deposition from the MTS-H_2_ gaseous system are extremely time-consuming and cost-prohibitive [30]. Fortunately, computational simulation technique has become a significant tool to explore this system. Simulation models with high-quality for the deposition process can be absolutely useful for optimizing the SiC deposition process [32]; such models may be coupling the fluid dynamics of the CVD reactor and the chemical kinetics of the growth process.

Molecular and/or radical reaction models [33,34,35,36,37,38], and thermodynamic models [30,37,39,40,41] have been proposed by several researchers for exploring the CVD process of SiC by MTS-H_2_ system; however, thorough kinetic mechanisms of both gas phase and surface reactions were absent in these works.

The kinetic mechanisms of H-Si-C-Cl gaseous system have been investigated by several researchers and could be employed for MTS-H_2_ system. Stefano Leone et al. [6] performed chemical kinetic analysis on the H-Si-C-Cl gaseous system using various Si, C, and Cl contained precursors, including MTS. Alessandro [25] and Fiorucci [42] reported kinetic mechanisms of surface reactions for H-Si-C-Cl system. For exploring MTS-H_2_ system, Kang Guan et al. [43,44] adopted a kinetic mechanism of gas phase reactions, including 74 gas phase reactions to investigate the CVD process of the epitaxial SiC deposition at 900~1400 °C, 6 kPa, and H_2_/MTS ratio of 3.4~4. Their works focus on developing a model which could reproduce the experimental results and employ numerical multiscale methodology in CVD processes of MTS-H_2_ system for SiC; however, their kinetic mechanism for surface reactions assumes that the gas phase species may adsorb to the vacancy bond of any Si or C atom that has been adsorbed, which may overestimate the adsorption efficiency of the gas phase species by overestimate the fraction of the open site on Si or C face.

Recently, Sukkaew and Danielsson [45,46,47] reported ab initio studies of adsorption and surface reactions of active C species and Si species by quantum chemical calculations, and proposed kinetic mechanisms of surface reactions for H-Si-C-Cl system; however, there are some adsorption kinetics of several intermediate species decomposing from MTS that are not contained in their mechanism.

Here, we discuss the applicability of different kinetic mechanisms of surface reactions for CVD modelling of epitaxial SiC proposed for H-Si-C-Cl system to the MTS-H_2_ gaseous system. Based on the kinetic mechanisms for surface reactions from H-Si-C-Cl system for epitaxial growth of SiC reported in Refs. [25,42,43,44,45,46,47], we proposed the surface kinetic mechanisms for MTS-H_2_ gaseous system. The component of site fraction of surface species and the growth rates are discussed, and the simplified mechanism with reduced surface reactions are reported.

## 2. Numerical Modeling

A horizontal hot-wall CVD reactor for SiC deposition is employed in this numerical simulation under low pressure and has been simplified in the 2D model. SiC deposition occurs along the substrate surface located on the susceptor in a growth chamber. The distance from the inlet of quartz tube to susceptor is approximately 450 mm. Deposition of the SiC film (4H SiC was considered) occurred on substrate surface.

Researchers explored the MTS-H_2_ gaseous system [30,37,39,40,41] by employment of computational thermodynamics, and their calculation results indicate that the phase stabilities of deposition highly depend on the process temperature and H_2_/MTS ratio. The ratio of H_2_/MTS in the range of 20~10^4^ will be beneficial for pure deposition of SiC [30,37], while the temperature in the range of 1027~1227 °C will be beneficial for optimum deposition [40]. Therefore, in this work, the process temperature employed above the substrate surface is around 1200 °C, and the ratio of H_2_/MTS is 30. The pressure employed in the reactor is 100 mbar.

**Figure 1:**
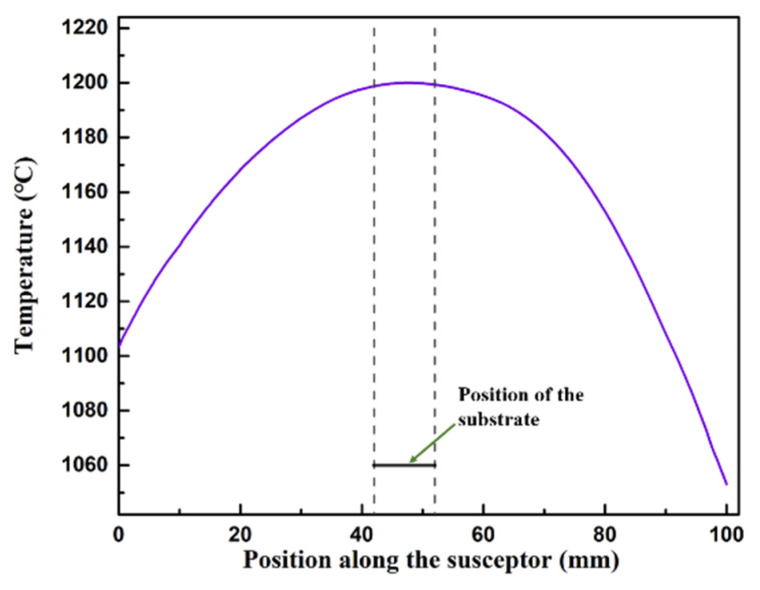
Temperature distribution along the susceptor.

The temperature on the susceptor surface keeps as fixed value for simplicity as shown in Figure 1. The temperature along the susceptor is not homogenous (~145 °C of variation). The maximum of temperature is around 1200 °C. The gas region upon the susceptor were in the temperature range of 1055~1200 °C under the deposition pressure of 100 mbar. The temperature on the position where the substrate located is ~1200 °C. The effects of reactions on temperature distribution and fluid flow were neglected.

The chemical kinetics of MTS-H_2_ gaseous system contained 74 reactions proposed by Kang Guan et al. [43,44] based on several sources of H-Si-C-Cl system [6,20,25,39,48], which kinetic and thermodynamic data are determined by quantum chemistry or experimental measurement, were employed in this model to calculate the rates of gas phase reactions. Their gas phase reaction model has been verified by the tail gas test [43].

Both chemical reaction kinetics and physical transfer phenomena are contained in our simulation. The calculated methods of heat and mass transfer, fluid flow and chemical kinetic mechanism in the gas phase were elaborated in our previous work [49] and are not repeated here. Assumptions in our calculations are as here: the gas mixture in the reactor is treated as an ideal gas, and the flow is assumed to be laminar due to the low Mach number [50]; the change of total volume of gas phase is neglected [31]; and surface reactions take place over the top surface of the substrate plate only [31].

Surface reactions are characterized by complicated reaction mechanisms. Adsorption reactions, desorption reactions, reactions between surface species, and growth reactions are contained. In the laminar flow model, transport of species toward the surface occurs mainly by diffusion through the fluid flow boundary layer [45]. The mass diffusion coefficient is

(1) $$D_{i}=\frac{1-X_{i}}{\sum_{j\neq i}^{\;}\frac{X_{j}}{D_{ij}}}$$

where $X_{i}$ is the molar fraction and $D_{ij}$ is the binary diffusion coefficient [45]. Here, considering the surface reaction of the following general form [14]:

(2) $$\overset{N_{g}}{\underset{i=1}{\sum }}a_{i}A_{i}\left(g\right)+\overset{N_{s}}{\underset{i=1}{\sum }}b_{i}B_{i}\left(s\right)+\overset{N_{b}}{\underset{i=1}{\sum }}c_{i}C_{i}\left(b\right)=\;\overset{N_{g}}{\underset{i=1}{\sum }}a_{i}^{’}A_{i}\left(g\right)+\overset{N_{s}}{\underset{i=1}{\sum }}b_{i}^{’}B_{i}\left(s\right)+\overset{N_{b}}{\underset{i=1}{\sum }}c_{i}^{’}C_{i}\left(b\right)$$

where g, s, and b in parentheses indicate gas-phase, surface, and bulk species, respectively [45], and $N_{g}$, $N_{s}$, $N_{b}$ are total numbers of gas-phase, surface, and bulk species, respectively [14]. The rate of the surface reaction is

(3) $$\dot{S}=R_{f}-R_{r}$$

where $R_{f}$ and $R_{r}$ are the forward and reverse reaction rate, respectively. Considering there is a surface reaction set including J reactions and I species, the rate of production of the ith species is

(4) $$r_{i}=\overset{J}{\underset{j=1}{\sum }}{\left(-1\right)}^{n}v_{ij{\dot{S}}_{j}}$$

where $v_{ij}$ is stoichiometric coefficient, ${\dot{S}}_{j}$ is the reaction rate of jth reaction, n is 1 when the species is as reactant or 0 when the species is as product.

The surface reactions from kinetic mechanisms, which investigated by using the density functional theory and transition state theory, proposed by Pitsiri Sukkaew et al. [45,46,47] for the H-Si-C-Cl system, are employed in the kinetic mechanism for surface reactions proposed in this work to calculate the growth rate of surface species on the epitaxial layer. Adsorption reactions, and reactions between adsorbed species are considered in this surface kinetic model, but effects of etching and doping are neglected; however, this mechanism could not accommodate well for the MTS-H_2_ gaseous system employed in this work because of the absence of consideration of several intermediate species. The other kinetic mechanism for surface reactions proposed for MTS-H_2_ system in this work is from the kinetic mechanisms proposed for H-Si-C-Cl system [25,42] with the employment of the sticking coefficient (SC) method for adsorption reactions reported in Refs. [43,44].

All the simulation steps were calculated by finite element method, using the commercial software COMSOL Multiphysics. In order to facilitate the generation of regular meshes to better the convergence of calculation, the substrate is assumed to be adhered to the susceptor [49].

## 3. Results and Discussion

This discussion will be divided into 3 parts. Firstly, we will focus on the composition of intermediate species above the substrate surface of gas-phase chemistry when using the kinetic mechanism for gas phase reactions of MTS-H_2_ system at 1200 °C and 100 mbar. Then the kinetic mechanism of surface reactions on the Si face (0001) of 4H SiC proposed by Pitsiri Sukkaew et al. [45,46,47] will be employed in the model for MTS-H_2_ gaseous system. In the last part, we proposed the kinetic mechanism of surface reactions from H-Si-C-Cl system [25,42] and simplified this mechanism without influencing the result of predicted growth rate.

As mentioned previously, most of the model conditions (i.e., boundary conditions of the simulation) were kept as a fixed value for the calculation. In our simulation, the total amount of each gas species (precursor and intermediate species) inside the reaction chamber can be calculated by chemical kinetics of gas phase reactions proposed in Refs. [43,44].

### 3.1. Gas Phase Reaction

The CVD process of 4H SiC is performed at around 1200 °C, with a pressure of around 100 mbar with the precursor, MTS, diluted in H_2_, the carrier gas, as mentioned previously. The H_2_/MTS ratio at the inlet of the tube is 30, and the gas flow rate of MTS is 20 sccm. Gas residence time can be estimated [43] as $\frac{V}{Q}\times \frac{T_{s}}{T}\times \frac{P}{P_{s}}$, where V, Q, $T_{s}$, $P_{s}$, T, P. are the effective reaction volume, gas flow rate, standard temperature, standard pressure, process temperature and pressure, respectively. In this model, the gas residence time is around 0.05 s, and is far smaller than the estimated thermal equilibrium time, beyond 1 s, reported in Refs. [43,51].

**Figure 2:**
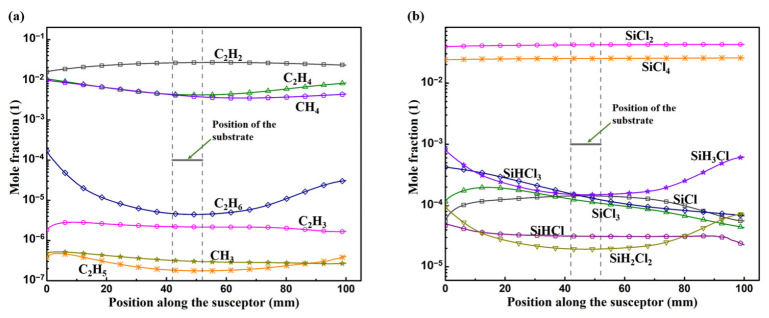
The mole fraction of intermediate species above the susceptor surface: (**a**) C contained species, and (**b**) Si contained species.

MTS decomposed in the reactor to different intermediate species, which contribute to the deposition of the epitaxial layer. Figure 2 shows the predicted mole fractions of C contained species and Si contained species above the substrate surface without consideration of surface reaction mechanism. C_2_H_2_ is the most abundant C contained species above the substrate surface while SiCl_2_ is the most abundant Si contained species. Besides, the concentration of SiCl_2_ and SiCl_4_ are stable above the susceptor with the temperature range of 1055~1200 °C. Mole fraction of C_2_H_6_ and SiH_3_Cl obviously decrease with the increasing temperature. CH and CH_2_ are also intermediates in this gas phase mechanism, but their fractions are too low, which are ~10^−12^ and ~10^−9^, respectively.

### 3.2. Surface Reactions on Si Face

From the kinetic mechanisms reported by Sukkaew and Danielsson [45,46,47] for H-Si-C-Cl system, considered that the species C_2_H_4_, C_2_H_2_, CH_4_, and CH_3_ are the active C species to the Si face, and the species SiHCl, SiCl, SiCl_2_, SiH, and Si are the active Si species to the adsorbed C species in this proposed kinetic mechanism for MTS-H_2_ system, and the reactions are listed in Table 1. Surface species included in these reactions are listed in Table 2.

It was assumed that the surface reactions occurred at the Si face (0001) of 4H SiC, which were terminated by two types of adsorption sites, with one type of site terminated by hydrogen atom (denoted by H(s)) while the other terminated by vacant site (denoted by O_Si_(s)) with an exposed dangling bond. By assuming that the surface sites have reached the equilibrium condition from RS1~RS4 in Table 1 at 1200 °C and 100 mbar, obtained the surface site fractions of H(s) and O_Si_(s) are about 0.3 and 0.7 on the Si face (0001).

Active C species, CH_4_, CH_3_, C_2_H_2_, and C_2_H_4_, would adsorb on both H(s) and O_Si_(s), and their sticking coefficients on both H(s) and O_Si_(s) are listed in Table 3. From RS5~RS12, assuming that the site fraction of H(s) and O_Si_(s) are 0.3 and 0.7 as mentioned previously, the evaluated adsorption rate for CH_4_, CH_3_, C_2_H_2_, and C_2_H_4_ on H(s) are around 2 × 10^−11^, 2 × 10^−7^, 1 × 10^−5^, and 1 × 10^−9^ molecule sites^−1^ s^−1^, respectively, while on O_Si_(s) are around 2 × 10^−4^, 3, 4 × 10^3^, and 3 × 10^2^ molecule sites^−1^ s^−1^, respectively, when the mole fraction of CH_4_, CH_3_, C_2_H_2_, and C_2_H_4_ assumed, as shown in Figure 2, to be ~4 × 10^−3^, ~3 × 10^−7^, ~2 × 10^−2^, and ~4 × 10^−3^, respectively; this will definitely overestimate the adsorption rates since the H(s) and O_Si_(s) would be consumed quickly in the reaction process.

**Figure 3:**
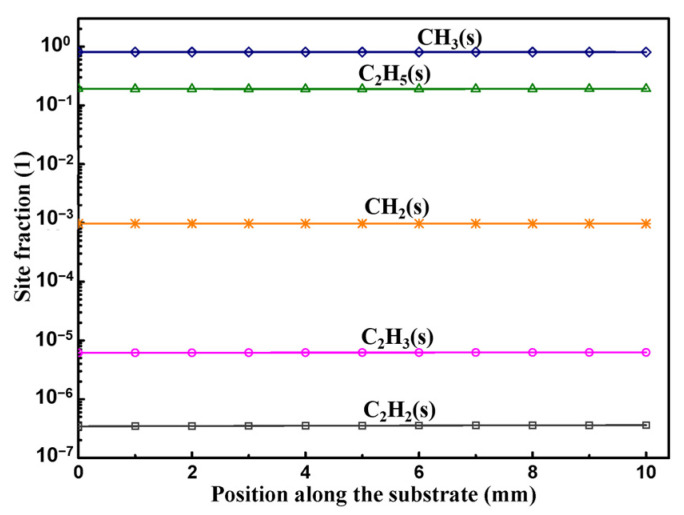
Site fraction of adsorbed C surface species on Si face.

Actually, with considering adsorption, desorption, and surface species reactions of active C species on Si face by reactions RS1~RS21 in Table. 1, the site fractions of adsorbed surface species at the equilibrium state greater than 10^−7^ are as shown in Figure 3. In this mechanism, CH_3_(s) is the most abundant active C surface species, about 80% of surface sites on Si face occupied by CH_3_(s). The site fraction of CH_3_(s) has been overestimated in this mechanism since the adsorption rate constant of the reaction RS12 from Ref. [45] has been overestimated by assuming the sticking coefficient of CH_3_ on O_Si_(s) equals to 1 and the reverse of RS9 and RS12 are not included here.

**Figure 4:**
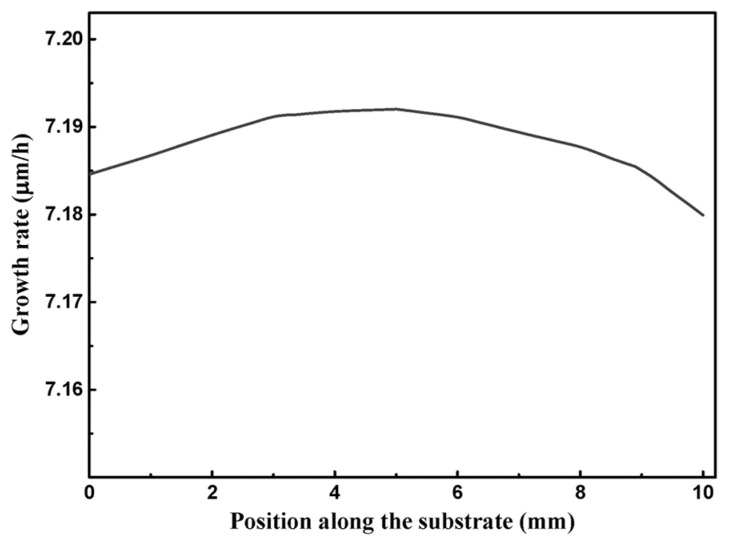
Predicted growth rate on the substrate by adsorption of Si and SiH.

From reactions RS22 ~ RS25, Si and SiH could contribute to the growth of epitaxial layer [45] by adsorbed on CH_2_(ads) and CH_3_(ads); however, neither Si nor SiH is the intermediate species of the MTS-H_2_ gas phase reaction mechanism employed in this model. Assumed that the mole fraction of Si and SiH above the substrate are ~10^−5^ and ~10^−6^, respectively, the predicted growth rate by reactions RS1~RS25 is as shown in Figure 4. The predicted growth rate is relatively lower than the reported data [52,53] of 4H SiC, about 170 μm/h under the temperature of 1600 °C by using MTS as the single precursor, or the reported data [44] of β-SiC about 18 μm/h under the temperature of 1200 °C by using MTS as the single precursor. Please note that, since there is no reported Si and SiH data when the temperature is 1200 °C and the ratio of H_2_/MTS is 30 in Ref. [30], or when the temperature range is in 900~1400 °C and the ratio range of H_2_/MTS is in 3.4~4 in Refs. [43,44], the employed values that we assumed for Si and SiH may be much larger than the actual ones, and would result in the overestimation of the predicted growth rate.

From reactions RS26 ~ RS33 in Table 1, the gas phase species SiCl, SiHCl, and SiCl_2_ could contribute to the growth of epitaxial layer [47] when they adsorbed on CH_3_(s) or C_2_H_4_(s), and the sticking coefficients are listed in Table. 3; however, these species could give feeble contribution to the growth rate. Even when we assume the fraction of CH_3_(s), without consideration of consumption by adsorption reactions of Si contained gas phase species, is about 0.8, the adsorption rate is ~2 × 10^−3^ molecule site^−1^ s^−1^ for SiCl adsorbed on CH_3_(s), and the growth rate for SiHCl and SiCl_2_ adsorbed on C_2_H_4_(s) are lower than ~10^−10^ molecule site^−1^ s^−1^, when the fraction of SiCl, SiHCl and SiCl_2_ assumed as shown in Figure 2, to be ~1 × 10^−4^, ~3 × 10^−5^, and ~4 × 10^−^^2^, respectively; their low adsorption rate in this mechanism can hardly contribute to the growth rate.

The surface reactions listed in Table 1 for the kinetic mechanism proposed in this work from the kinetic mechanisms for H-Si-C-Cl system reported in the investigations [45,46,47] could not contribute reasonable predicted growth rate for MTS-H_2_ system of SiC epitaxial process at 1200 °C and 100 mbar. In this surface kinetic mechanism, there are 4 C contained intermediate species considered as active to the Si face, while other 3 C contained intermediate species which mole fraction greater than 10^−7^ as shown in Figure 2 are not included. The Si contained intermediate species SiHCl, SiCl, and SiCl_2_ could merely contribute almost negligible growth rate on the adsorbed C face with their extremely low sticking coefficients on CH_3_(s) or C_2_H_4_(s). Besides, the probability of other Si contained intermediate species in Figure 2 to the adsorbed C species is not included in this kinetic mechanism.

### 3.3. Surface Reactions on Si Face

By employing the kinetic mechanisms proposed for H-Si-C-Cl system in Refs. [25,42] and sticking coefficient (SC) method reported in Refs. [43,44], herein, we proposed the kinetic mechanism of surface reactions for the MTS-H_2_ system, whilst the reactions involved are listed in Table 4.

The adsorption reactions for H-Si-C-Cl system proposed in the investigations [25,42,43,44] are in the form:

Si species + O_C_(s) → product(s), for Si species adsorption on the C face;

or C species + O_Si_(s) → product(s), for C species adsorption on the Si face.

O_C_(s) and O_Si_(s) present the open surface site on the C face and Si face, respectively; however, as mentioned previously, not every Si/C atom has an open surface site.

It was considered that the species C_2_H_6_, C_2_H_5_, C_2_H_4_, C_2_H_3_, C_2_H_2_, CH_4_, CH_2_, and CH are the active C species to the open site on Si face, and the species SiHCl_3_, SiH_3_Cl, SiH_2_Cl_2_, SiHCl, SiCl_4_, SiCl_3_, SiCl_2_, SiCl, and HCl are the active Si/Cl species to the open site on Si/C face. Silicon-rich and carbon-rich portions of the lattice are not considered here. Again, it was assumed that the deposition started on the Si face, and the open site on the C face existed on the adsorbed C species. The total active surface site fraction in this mechanism is about 0.7 on the Si face, as we discussed previously in Section 3.2, since the mechanism of reactions on the sites terminated by H atom on the Si face was not included here.

**Figure 5:**
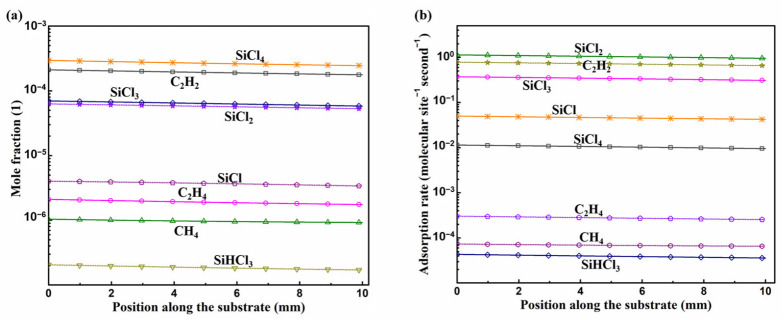
(**a**) Mole fraction and (**b**) adsorption rate of Si/C contained intermediate species on the substrate surface.

**Figure 6:**
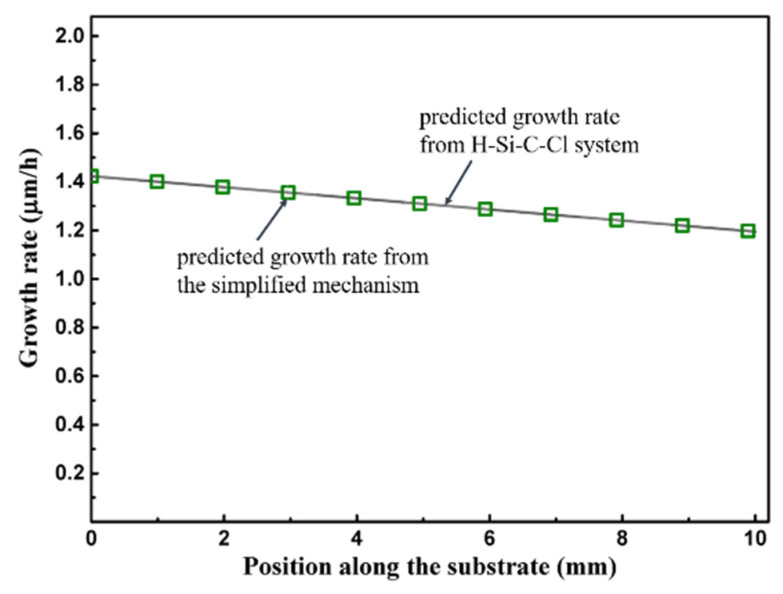
Predicted growth rate on the substrate surface.

With the effect of consumption of intermediate species above substrate surface by surface reactions, the mole fraction of Si/C contained intermediates above substrate surface which greater than 10^−7^ as shown in Figure 5a. These intermediate species contribute greatly to the growth rate and their adsorption rates are as shown in Figure 5b. Please note that the calculated adsorption rates here are by employing the sticking coefficients proposed in Refs. [43,44] for β-SiC. The mole fraction, of C_2_H_3_ and SiHCl is ~10^−8^, of SiH_3_Cl, and SiH_2_Cl_2_ is ~10^−9^, of C_2_H_5_ and C_2_H_6_ is ~10^−10^, and is lower than 10^−10^ for CH and CH_2_. The intermediate SiHCl has a relatively higher contribution to the growth rate since its adsorption rate is ~10^−3^ molecular site^−1^ s^−1^. The adsorption rates of other intermediate species, which mole fraction lower than 10^−7^, could merely contribute inappreciably to the deposition, of which are lower than 10^−4^ molecule site^−1^ s^−1^ for C_2_H_3_ and SiH_3_Cl, lower than 10^−6^ molecule site^−1^ s^−1^ for CH_2_, C_2_H_5_ and SiH_2_Cl_2_, and lower than 10^−8^ molecule site^−1^ s^−1^ for CH and C_2_H_6_. Therefore, the surface reactions listed in Table 4 for MTS-H_2_ system from H-Si-C-Cl system [25,42,43,44] can be reduced by removing adsorption reactions which contribute weakly to the growth rate and reactions which rate constants lower than 10^−7^ molecular site^−1^ s^−1^ without influencing the predicted growth rate by this mechanism, as illustrated in Figure 6, and the reactions of simplified mechanism are listed in Table 5. The predicted growth rate is also relatively low here. When considering the H_2_/MTS to be 4 here by employing this mechanism, the predicted growth rate would be about 8.5 μm/h, and it could be the reasonable value when compare to the predicted growth rate, 18 μm/h, reported in Ref. [44] with its deposition temperature is 1200 ℃ and H_2_/MTS is 3.4; this kinetic mechanism proposed here has the same property as the mechanisms proposed in Refs. [25,42], that is, the predicted growth rate would be decreased with increased H_2_/MTS ratio. Due to this property of the mechanism that we proposed here, the predicted growth rate may be underestimated when the H_2_/MTS is relatively high. Besides, the lack of reaction mechanism for the surface sites on Si face terminated by H atom also results in the underestimation of the predicted growth rate.

## 4. Conclusions

In this work, we proposed kinetic mechanisms for the MTS-H_2_ system from kinetic mechanisms for the H-Si-C-Cl system for epitaxial silicon carbide deposition. It was assumed that the deposition started on the Si face where the surface site was terminated by open site or H atom. The first kinetic mechanism proposed in this work could not contribute a reasonable predicted growth rate for MTS-H_2_ system of SiC epitaxial process because there are several adsorption reactions of intermediate species in the gas phase of MTS-H_2_ gaseous system that are not contained in this mechanism, especially considering that the mechanisms of the adsorption of Si species on the adsorbed C surface species are unclear. The second kinetic mechanism and its simplified mechanism proposed in this work could underestimate the predicted growth rate, since its properties and the lack of reaction mechanism for the surface sites on the Si face are terminated by H atom; however, these mechanisms are still valuable and need to be improved.

## Figures and Tables

**Table 1:** Kinetic mechanism of surface reaction for SiC.

		Forward Rate Constant	Reverse Rate Constant
		1200 ℃	1200 ℃
	**Surface site equilibrium reactions**		
RS1	H(g) + H(s) → H_2_(g) + O_Si_(s)	6.75 × 10^7 a,#^	-
RS2	H_2_(g) + O_Si_(s) → H(g) + H(s)	5.29 × 10^4 a,#^	-
RS3	H (g) + O_Si_(s) → H(s)	1.29 × 10^8 c,#^	-
RS4	H(s) + H(s) → H_2_(g) + 2O_Si_(s)	1.25 × 10^5 c,@^	-
	**Adsorption reactions of active C species on the Si surface**		
RS5	CH_3_(g) + H(s) → CH_3_(s) + H(g)	2.45 ^a,#^	-
RS6	CH_4_(g) + H(s) → CH_3_(s) + H_2_(g)	2.13 × 10^−8 a,#^	-
RS7	C_2_H_2_(g) + H(s) → C_2_H_3_(s)	2.4 × 10^−3 a,#^	-
RS8	C_2_H_4_(g) + H(s) → C_2_H_5_(s)	1.25 × 10^−6 a,#^	-
RS9	CH_4_(g) + O_Si_(s) → CH_3_(s) + H(g)	5.58 × 10^−2 a,#^	-
RS10	C_2_H_2_(g) + O_Si_(s) → C_2_H_2_(s)	3.02 × 10^5 a,#^	-
RS11	C_2_H_4_(g) + O_Si_(s) → C_2_H_4_(s)	1.2 × 10^5 a,#^	-
RS12	CH_3_(g) + O_Si_(s) → CH_3_(s)	1.4 × 10^7 c,#^	-
RS13	CH_3_(s) + H(g) → CH_2_(s) + H_2_(g)	1.7 × 10^5 a,#^	-
RS14	CH_2_(s) + H_2_(g) → CH_3_(s) + H(g)	6.7 × 10^2 a,#^	-
RS15	H(g) + CH_2_(s) → CH_3_(s)	1.29 × 10^8 c,#^	-
RS16	H(g) + CH(s)-CH_2_(s) → CH_2_(s) + CH_2_(s)	1.29 × 10^8 c,#^	-
	**Surface species reactions on the Si surface**		
RS17	CH_2_(s) + H(s) → CH_3_(s) + O_Si_(s)	6.32 × 10^8 b,@^	-
RS18	C_2_H_2_(s) + H(s) → C_2_H_3_(s) + O_Si_(s)	1.9 × 10^11 b,@^	-
RS19	C_2_H_3_(s) + O_Si_(s) → CH(s)-CH_2_(s)	3.54 × 10^9 b,@^	-
RS20	C_2_H_4_(s) + H(s) → C_2_H_5_(s) + O_Si_(s)	6.83 × 10^10 b,@^	-
RS21	C_2_H_5_(s) + O_Si_(s) → CH_2_(s) + CH_3_(s)	1.09 × 10^4 b,@^	-
	**Growth reactions**		
RS22	Si(g) + CH_2_(s) → H_2_(g) + O_Si_(s) + SiC(b)	2.36 × 10^7 c,#^	-
RS23	Si(g) + CH_3_(s) → H_2_(g) + H(s) + SiC(b)	4 × 10^6 c,#^	-
RS24	SiH(g) + CH_2_(s) → H_2_(g) + H(s) + SiC(b)	2.32 × 10^7 c,#^	-
RS25	SiH(g) + CH_3_(s) → H_2_(g) + H(g) + H(s) + SiC(b)	1.15 × 10^3 c,#^	-
	**Adsorption reactions of active Si species on the C surface**		
RS26	SiCl(g) + CH_3_(s) → SiHCl-CH_2_(s)	2.63 × 10^1 a,#^	-
RS27	SiHCl(g) + C_2_H_4_(s) + O_Si_(s) → SiHCl-(CH_2_)_2_(s)	1.03 × 10^−4 a,#^	-
RS28	SiCl_2_(g) + C_2_H_4_(s) + O_Si_(s) → SiCl_2_-(CH_2_)_2_(s)	4.44 × 10^−6 a,#^	-
	**Surface species reactions on the C surface**		
RS29	SiHCl-CH_2_(s) + CH_3_(s) → SiHCl-(CH_2_)_2_(s) + H(g)	2.74 × 10^5 b,@^	-
RS30	SiHCl-(CH_2_)_2_(s) + CH_3_(s) → SiH-(CH_2_)_3_(s) + HCl(g)	1.37 × 10^4 b,@^	4.65 × 10^−1 b,#^
RS31	SiHCl-(CH_2_)_2_(s) + CH_3_(s) → SiCl-(CH_2_)_3_(s) + H_2_(g)	1.84 × 10^3 b,@^	1.24 × 10^−4 b,#^
	**H atom abstraction reactions**		
RS32	SiH-(CH_2_)_3_(s) + H(g) → Si-(CH_2_)_3_(s) + H_2_(g)	2.11 × 10^8 b,#^	1.48 × 10^5 b,#^
RS33	SiCl-(CH_2_)_3_(s) + H(g) → Si-(CH_2_)_3_(s) + HCl(g)	1.35 × 10^5 b,#^	4.8 × 10^4 b,#^

The rate constant is in the unit of molecule site^−1^ s^−1^, g in parentheses indicates gas phase species, s in parentheses indicates surface species, b in parentheses indicates solid species, O_Si_(s) present Si surface site; ^a^ rate constant is calculated by $A_{s}S_{t}\frac{P}{\sqrt{2\pi mk_{B}T}}$ from Refs. [46,47], where $A_{s}$, $S_{t}$, m, $k_{B}$, T, and P refer, respectively, to the area per one site on the Si face, the sticking coefficient, the mass of the gas phase species above the substrate, the Boltzmann constant, temperature (1200 ℃) and total pressure (100 mbar); ^b^ the rate constant is calculated from the expression and its terms reported in Refs. [46,47], and ^c^ the rate constant is calculated from the expression and its terms reported in Ref. [45]; ^#^ the reaction rate calculated by $k\overset{N_{i}}{\underset{i=1}{\prod }}\gamma_{i}^{v_{i}}\overset{N_{j}}{\underset{j=1}{\prod }}\varphi_{j}^{v_{j}}$, and ^@^ the reaction rate calculated by $k\overset{N_{j}}{\underset{j=1}{\prod }}\varphi_{j}^{v_{j}}$, where k is the rate constant, $N_{i}$ and $N_{j}$ are the number of gas phase species and surface species, $\gamma_{i}$ is the mole fraction of gas phase species i, $\varphi_{j}$ is the fraction of surface species j, $v_{i}$ and $v_{j}$ are the stoichiometric coefficients for gas phase species i and surface species j.

**Table 2:** Surface species occupy 1 surface site or 2/3 surface sites on Si face.

1 Surface Site Occupied	2 Surface Sites Occupied	3 Surface Sites Occupied
H(s), CH_2_(s), CH_3_(s), C_2_H_2_(s), C_2_H_3_(s), C_2_H_4_(s), C_2_H_5_(s), SiHCl-CH_2_(s)	CH(s)-CH_2_(s), SiCl_2_-(CH_2_)_2_(s), SiHCl-(CH_2_)_2_(s)	SiCl-(CH_2_)_3_(s), SiH-(CH_2_)_3_(s), Si-(CH_2_)_3_(s)

s in parentheses indicates surface species. The area per one surface site is assumed be 8.178 × 10^−^^20^ m^2^ on Si face of 4H SiC.

**Table 3:** Sticking coefficient of intermediate species on surface site or adsorbed species.

	Sticking Coefficient			
	On H(s)	On O_Si_(s)	On CH_3_(s)	On C_2_H_4_(s)
CH_3_	1.7 × 10^−7 a^	1 ^c^	-	-
CH_4_	1.5 × 10^−15 a^	4 × 10^−9 a^	-	-
C_2_H_2_	2.2 × 10^−10 a^	2.8 × 10^−2 a^	-	-
C_2_H_4_	1.2 × 10^−13 a^	1.1 × 10^−2 a^	-	-
SiCl	-	-	3.7 × 10^−6 b^	-
SiHCl	-	-	-	1.5 × 10^−11 b^
SiCl_2_	-	-	-	7.9 × 10^−13 b^

The sticking coefficient is estimated from the expression and its terms in ^a^ Ref. [46] and the ^b^ Ref. [47] at 1200 ℃ and 100 mbar, ^c^ the sticking coefficient is assumed.

**Table 4:** Surface reaction mechanism for SiC from H-Si-C-Cl system.

	**Adsorbed Reactions of Active C Species**	Sticking Coefficient ^a^
RE1	CH(g) + O_Si_(s) → CH(s)	0.01
RE2	CH_2_(g) + O_Si_(s) → C(s) + H_2_(g)	0.01
RE3	CH_4_(g) + O_Si_(s) → C(s) + 2H_2_(g)	5 × 10^−5^
RE4	C_2_H_2_(g) + 2O_Si_(s) → 2C(s) + H_2_(g)	0.02
RE5	C_2_H_3_(g) + 2O_Si_(s) → C(s) + CH(s) + H_2_(g)	0.03
RE6	C_2_H_4_(g) + 2O_Si_(s) → 2C(s) + 2H_2_(g)	0.0016
RE7	C_2_H_5_(g) + 2O_Si_(s) → C(s) + CH(s) + 2H_2_(g)	0.03
RE8	C_2_H_6_(g) + 2O_Si_(s) → 2C(s) + 3H_2_(g)	0.0016
	**Adsorbed reactions of active Si/Cl species**	Sticking coefficient ^a^
RE9	SiHCl_3_(g) + 2O_Si_(s) + 2O_C_(s) → SiCl(s) + H(s) + 2Cl_Si_(s)	0.01
RE10	SiHCl_3_(g) + O_Si_(s) + 3O_C_(s) → SiCl(s) + H(s) + Cl_Si_(s) + Cl_C_(s)	0.01
RE11	SiH_3_Cl(g) + 2O_C_(s) → SiCl(s) + H(s) + H_2_(g)	0.01
RE12	SiH_2_Cl_2_(g) + O_Si_(s) + 3O_C_(s) → SiCl(s) + 2H(s) + Cl_Si_(s)	0.01
RE13	SiHCl(g) + O_C_(s) → Si(s) + HCl(g)	0.02
RE14	SiCl_4_(g) + 2O_Si_(s) + 2O_C_(s) → SiCl(s) + Cl_C_(s) + 2Cl_Si_(s)	0.01
RE15	SiCl_3_(g) + O_Si_(s) + 2O_C_(s) → SiCl(s) + Cl_C_(s) + Cl_Si_(s)	0.02
RE16	SiCl_3_(g) + 3O_C_(s) → SiCl(s) + 2Cl_C_(s)	0.02
RE17	SiCl_3_(g) + 2O_Si_(s) + O_C_(s) → SiCl(s) + 2Cl_Si_(s)	0.02
RE18	SiCl_2_(g) + O_Si_(s) + O_C_(s) → SiCl(s) + Cl_Si_(s)	0.02
RE19	SiCl_2_(g) + 2O_C_(s) → SiCl(s) + Cl_C_(s)	0.02
RE20	SiCl(g) + O_C_(s) → SiCl(s)	0.01
RE21	HCl(g) + O_Si_(s) + O_C_(s) → H(s) + Cl_Si_(s)	0.02
RE22	HCl(g) + 2O_C_(s) → H(s) + Cl_C_(s)	0.02
	**Cl abstraction reactions**	Rate constant ^#^
RE23	HCl(g) + SiCl(s) → SiCl_2_(g) + H(g) + O_C_(s)	1.34 × 10^6 c^
RE24	Cl_C_(s) + H(g) → HCl(g) + O_C_(s)	1.19 × 10^8 b^
RE25	Cl_Si_(s) + H(g) → HCl(g) + O_Si_(s)	1.19 × 10^8 b^
RE26	2Cl_C_(s) + SiCl_2_(g) → SiCl_4_(g) + 2O_C_(s)	3 × 10^−5 b^
RE27	2Cl_C_(s) + H_2_(g) → 2HCl(g) + 2O_C_(s)	1.22 × 10^−10 c^
RE28	2Cl_Si_(s) + H_2_(g) → 2HCl(g) + 2O_Si_(s)	5.96 × 10^−12 c^
RE29	Cl_Si_(s) + Cl_C_(s) + H_2_(g) → 2HCl(g) + O_Si_(s) + O_C_(s)	2.69 × 10^−11 b^
	**Surface species reactions**	Rate constant ^@^
RE30	SiCl(s) + Cl_C_(s) → SiCl_2_(g) + 2O_C_(s)	9.18 × 10^7 b^
RE31	SiCl(s) + Cl_Si_(s) → SiCl_2_(g) + O_C_(s) + O_Si_(s)	6.8 × 10^−1 b^
RE32	2SiCl(s) → SiCl_2_(g) + Si(s) + O_C_(s)	6.8 × 10^−1 b^
RE33	SiCl(s) + H(s) → HCl(g) + Si(s) + O_C_(s)	2.06 × 10^1 b^
RE34	Si(s) + Cl_Si_(s) → SiCl(s) + O_Si_(s)	2.03 × 10^8 b^
RE35	Si(s) + Cl_C_(s) → SiCl(s) + O_C_(s)	2.03 × 10^8 b^
RE36	Cl_Si_(s) + H(s) → HCl(g) + O_Si_(s) + O_C_(s)	6.76 × 10^3 b^
RE37	Cl_C_(s) + H(s) → HCl(g) + 2O_C_(s)	3.05 × 10^4 b^
RE38	H(s) + H(s) → H_2_(g) + 2O_C_(s)	1.55 × 10^8 b^
	**Growth reactions**	Rate constant ^@^
RE39	SiCl(s) + C(s) → SiC(b) + Cl(g) + O_C_(s) + O_Si_(s)	2.03 × 10^8 b^
RE40	Si(s) + C(s) → SiC(b) + O_C_(s) + O_Si_(s)	2.03 × 10^8 b^
RE41	SiCl(s) + CH(s) → SiC(b) + HCl(g) + O_C_(s) + O_Si_(s)	2.03 × 10^8 b^
RE42	Si(s) + CH(s) → SiC(b) + H(g) + O_C_(s) + O_Si_(s)	2.03 × 10^8 b^

The rate constant is in the unit of molecule site^−1^ s^−1^, g in parentheses indicates gas phase species, s in parentheses indicates surface species, b in parentheses indicates solid species, O_Si_(s) and O_C_(s) present Si and C surface site, subscripts Si and C present surface species on Si and C surface site; ^a^ the reaction rate is calculated from the Sticking coefficient (SC) method reported in Ref. [43], ^b^ the rate constant is calculated from the expression and its terms reported in Ref. [25], and ^c^ the rate constant is calculated from the expression and its terms reported in Ref. [42]; ^#^ the reaction rate calculated by $k\overset{N_{i}}{\underset{i=1}{\prod }}\gamma_{i}^{v_{i}}\overset{N_{j}}{\underset{j=1}{\prod }}\varphi_{j}^{v_{j}}$, and ^@^ the reaction rate calculated by $k\overset{N_{j}}{\underset{j=1}{\prod }}\varphi_{j}^{v_{j}}$, where k is the rate constant, $N_{i}$ and $N_{j}$ are the number of gas phase species and surface species, $\gamma_{i}$ is the mole fraction of gas phase species i, $\varphi_{j}$ is the fraction of surface species j, $v_{i}$ and $v_{j}$ are the stoichiometric coefficients for gas phase species i and surface species j. In this kinetic mechanism, surface species adsorbed on Si face including CH(s), C(s), and Cl_Si_(s); surface species adsorbed on C face including Si(s), SiCl(s), Cl_C_(s), and H(s).

**Table 5:** Simplified kinetic mechanism for surface reactions.

CH_4_(g) + O_Si_(s) → C(s) + 2H_2_(g)	C_2_H_2_(g) + 2O_Si_(s) → 2C(s) + H_2_(g)
C_2_H_4_(g) + 2O_Si_(s) → 2C(s) + 2H_2_(g)	H(s) + H(s) → H_2_(g) + 2O_C_(s)
SiHCl_3_(g) + 2O_Si_(s) + 2O_C_(s) → SiCl(s) + H(s) + 2Cl_Si_(s)	SiHCl_3_(g) + O_Si_(s) + 3O_C_(s) → SiCl(s) + H(s) + Cl_Si_(s) + Cl_C_(s)
SiHCl(g) + O_C_(s) → Si(s) + HCl(g)	SiCl_4_(g) + 2O_Si_(s) + 2O_C_(s) → SiCl(s) + Cl_C_(s) + 2Cl_Si_(s)
SiCl_3_(g) + O_Si_(s) + 2O_C_(s) → SiCl(s) + Cl_C_(s) + Cl_Si_(s)	SiCl_3_(g) + 3O_C_(s) → SiCl(s) + 2Cl_C_(s)
SiCl_3_(g) + 2O_Si_(s) + O_C_(s) → SiCl(s) + 2Cl_Si_(s)	SiCl_2_(g) + O_Si_(s) + O_C_(s) → SiCl(s) + Cl_Si_(s)
SiCl_2_(g) + 2O_C_(s) → SiCl(s) + Cl_C_(s)	SiCl(g) + O_C_(s) → SiCl(s)
HCl(g) + O_Si_(s) + O_C_(s) → H(s) + Cl_Si_(s)	HCl(g) + 2O_C_(s) → H(s) + Cl_C_(s)
HCl(g) + SiCl(s) → SiCl_2_(g) + H(g) + O_C_(s)	Cl_C_(s) + H(g) → HCl(g) + O_C_(s)
Cl_Si_(s) + H(g) → HCl(g) + O_Si_(s)	2Cl_C_(s) + SiCl_2_(g) → SiCl_4_(g) + 2O_C_(s)
SiCl(s) + Cl_C_(s) → SiCl_2_(g) + 2O_C_(s)	SiCl(s) + Cl_Si_(s) → SiCl_2_(g) + O_C_(s) + O_Si_(s)
2SiCl(s) → SiCl_2_(g) + Si(s) + O_C_(s)	SiCl(s) + H(s) → HCl(g) + Si(s) + O_C_(s)
Si(s) + Cl_Si_(s) → SiCl(s) + O_Si_(s)	Si(s) + Cl_C_(s) → SiCl(s) + O_C_(s)
Cl_Si_(s) + H(s) → HCl(g) + O_Si_(s) + O_C_(s)	Cl_C_(s) + H(s) → HCl(g) + 2O_C_(s)
SiCl(s) + C(s) → SiC(b) + Cl(g) + O_C_(s) + O_Si_(s)	Si(s) + CH(s) → SiC(b) + H(g) + O_C_(s) + O_Si_(s)
Si(s) + C(s) → SiC(b) + O_C_(s) + O_Si_(s)	Si(s) + C(s) → SiC(b) + O_C_(s) + O_Si_(s)
SiCl(s) + CH(s) → SiC(b) + HCl(g) + O_C_(s) + O_Si_(s)	
